# Prevalence of psychotic disorders in an urban area of France

**DOI:** 10.1186/s12888-015-0588-5

**Published:** 2015-08-25

**Authors:** Andrei Szöke, Grégoire Baudin, Ghassen Saba, Baptiste Pignon, Jean-Romain Richard, Marion Leboyer, Franck Schürhoff

**Affiliations:** AP-HP, DHU PePSY, Groupe Hospitalier “Mondor”, Pôle de Psychiatrie, Créteil, 94000 France; INSERM (French National Institute of Health and Medical Research), U955, team 15, Créteil, 94000 France; UPEC, University Paris-Est, Faculté de médecine, Créteil, 94000 France; Fondation FondaMental, Créteil, 94000 France; University François-Rabelais of Tours, PAV EA 2114, Tours, 37000 France; CHRU de Lille, Department of Psychiatry, Fontan Hospital, Lille, 59000 France; Pôle de Psychiatrie, Hôpital “A. Chenevier”, Pavillon Hartmann, 40, rue de Mesly, Créteil, 94000 France

## Abstract

**Background:**

Most data on the prevalence of psychotic disorders is limited to global estimates or restricted to schizophrenia. Consequently, there is limited information available about the prevalence of psychotic disorders more widely and outwith age and sex - specific prevalence values. The objective of this study is to provide period prevalence estimates, detailed by gender and age groups, for treated psychotic disorders in an adult population (aged 18 years and over) from an urban area in France.

**Methods:**

Prospective reporting of cases treated over an 8-week period complemented by several methods estimating the number of potentially missed cases, including a leakage study. The study took place in an urban, well defined catchment area, with a population of 67 430 at risk subjects living in the east of a Paris suburb.

**Results:**

The observed prevalence was of 3.72 per 1000 subjects at risk; after adjustment for potentially lost cases the estimate was of 4.60 per 1000 subjects at risk. Observed prevalence was higher in men (4.71 per 1000, Relative Risk = 1.68) and in the 35–45 age-band (6.05 per 1000, Relative Risk = 1.93).

**Conclusion:**

Global prevalence estimates of psychotic disorders in this study are in line with expected values based on studies conducted in other countries. Careful consideration of the causes of missed cases and gathering of complementary data are essential and could result in significant changes in prevalence estimates. Detailed estimates (by age) suggest that treated psychosis might not be a lifelong condition.

## Background

Psychotic disorders, characterized by delusions and/or hallucinations, are the most severe of all mental disorders, representing a major burden for the individual and the society as well as constituting one of the major causes of years lived with disability (YLD) in Europe [1]. Prevalence data provide an estimate of the burden of disease for society and can be used to inform resource allocation and mental health policy [2, 3].

The prevalence of a disorder is the proportion of people, in a community, who have the disorder at a given time (point prevalence), over a given period of time (period prevalence) including both pre-existing disease and those who newly develop the disease over this period or who have ever had the disorder (life-time prevalence) including people that are in remission.

There are two main methods to identify cases for prevalence estimation: clinical studies that enumerate cases in the health system (including local or national case-registries) and population surveys. No method is perfect and each of them has advantages and disadvantages.

Clinical studies are usually less expensive, simpler to implement, and can provide more detailed data [4]. However, clinical studies typically do not account for cases that are not in the health system. The probability of being in treatment can depend on several factors not related to the burden of morbidity, such as availability of services, their location and accessibility and the rate of their utilisation. The extent of selection bias due to these factors is probably less important for severe disorders such as schizophrenia [5]. Case-registries are systematic, cumulative databases that improve exhaustivity. They have advantages: coverage of a defined population, cumulative case registration over long periods of time and with the capacity to link cases with records/information from other databases. However, they are unable to take into account cases that never come up for assessment or treatment, as well as providing less detailed clinical information and have problems which include diagnostic validity [6].

On the other hand, population surveys can offer information on subjects that are not in contact with the health system. However, they are more difficult to implement and not free of possible bias, primarily mainly due to non-responders, which could preferentially be among the subjects with the disorder being surveyed. Furthermore, for rare mental health disorders, such as schizophrenia, the validity of the information can be limited [4], due to difficulties in achieving large enough samples. As such, the different methods of enumerating cases can be seen as offering complementary information on prevalence. Prevalence data provide an estimation of the disease burden on a society and, by assessing needs and service utilisation, can be used to inform resource allocation and mental health policy [2, 4].

In 2005, Saha and colleagues conducted the largest systematic review of the prevalence of schizophrenia to date. They included data from 188 studies conducted in 46 countries. The median period prevalence value per 1000 persons was 3.3 (80 % confidence interval ranging from 1.3 to 8.2 per 1000). However, questions as to the generalizability of this data emerge, given that most of these observations come from a very small number of countries. Of the 132 core studies identified by Saha and colleagues, more than half were conducted in only 4 countries (United States, United Kingdom, India and Canada). A similar question as to the generalizability also occurs in data from Europe, where more than 25 % of the studies came from the United Kingdom, with 50 % coming from only three countries (United Kingdom, Denmark and Germany) [7]. Although the study by Saha and colleagues was a thorough review, some comparisons of interest, between specific populations, are not available due to the lack of original data. For example, there are no prevalence figures according to age categories, even although such data combined with incidence data could add to our understanding of the outcome (e.g. mean duration of the disorder, remission rates) [8].

Furthermore, data from prevalence studies which looked at psychotic disorders more widely, and not limited to schizophrenia alone, are very scarce [9–11]. Prevalence figures for psychotic disorders are variable (ranging from 0.7 to 0.9 % in the cited studies), as is the proportion of cases diagnosed with schizophrenia (ranging from 0.3 to 0.9 %), mainly due to variations in the definition of "psychotic disorders".

To our knowledge, there are only a few studies in France that have investigated the prevalence of psychotic disorders [12–16]. Some of these studies were in specific populations (e.g. Falissard et al. studied a population of male inmates, finding 3.8 % to have psychotic disorders [16]) or in particular settings (e.g. Jay et al. found very high prevalence rates, 1.49 %, in the Réunion Island - a French overseas region in the Indian Ocean [14]). However, given the specificities of these populations, the figures are not representative of prevalence of psychoses in the general French population. In the general French population, there have been 3 studies to date. Brunetti and colleagues investigated the prevalence of mental disorders in a rural town of around 800 inhabitants in 1975. The prevalence of psychotic disorders (period-prevalence: one year) was estimated at between 1 and 2 % of the population but, given the size of the at risk population, it is difficult to generalize from these findings [12]. According to the study by Sadoun and colleagues, the point prevalence of hospitalized patients in France was of 0.61 to 0.79 per 1000 for schizophrenia and 0.88 to 1.19 %, when other psychotic disorders were included [13]. More recently, the French Mental Health in General Population (MHGP) survey, conducted by the World Health Organization Collaborating Centre (WHO-CC), between 1999 and 2003, included more than 37000 participants, that were representative of the French population. Subjects were interviewed using the Mini International Neuropsychiatric Interview (MINI) [17] in 47 sites across France, with a life-time psychotic disorder being diagnosed in 2.7 % of the sample (2 % recurrent psychotic disorders and 0.7 % single psychotic episodes) [15, 18]. As such, data on psychosis prevalence is very limited in France, with no recent estimates and large differences in prevalence from the limited previous studies.

In order to obtain prevalence data on psychosis in France, we conducted a study estimating the period-prevalence of treated psychosis in an urban area of France, detailed by gender and age-bands. Special attention was given to the precision of the estimates by limiting the number of unidentified cases and by identifying and correcting all possible sources of lost cases.

## Methods

### Catchment area

The study took place in the biggest town of the eastern suburb of Paris, Créteil (Val de Marne county). The population of this town, according to the most recent census (2011), is of 90 528 (67 430 aged 18 years and over). Créteil is a densely populated area with 7839.9 inhabitants per square kilometre (versus 990.5 in Ile-de France region, and 117.0 in the whole of mainland France), with an ethnically diverse population (migrants represent 23.48 % of the population compared to 18.16 % for the whole Region and 8.84 % for mainland France) and a high unemployment rate: 14.5 % (versus 8.6 %, and 9.4 % respectively) [19].

In France, most of the patients diagnosed with severe psychiatric disorders are treated by psychiatrists within public practice. A minority are treated by psychiatrists in private practice and by general practitioners (GP). The public sector is organized in catchment areas ("secteurs psychiatriques") that cover well-defined areas of 60000 to 80000 inhabitants. Each of these catchment areas has inpatient and outpatient settings. For example, the Creteil psychiatric "secteur" has 6 inpatient units and several outpatient units (for consultations, day care centre, cognitive remediation, etc.).

### Participants

We compiled a list of all the practitioners (psychiatrists and GP) who worked in Creteil and potentially followed psychotic patients, using several sources (administrative sources, Yellow Pages, internet search, etc.). We tried to contact each of these physicians by phone, mail and finally, when needed, met with them at their office.

When contact was established, the study rationale and methodology were explained to each practitioner who was asked to participate. When the physician declined, a request was made to give an estimation of the mean number of adult patients with psychotic disorders for which they prescribed an antipsychotic treatment, over an 8-week period. There were two reasons for asking for this estimation. The first was to be able to compare - in terms of the (expected) number of treated psychotic subjects - the practitioners that participated in the whole study with those who did not. Secondly, these numbers were used to estimate the number of patients treated by the practitioners that did not participate in the whole study (for details see below, in additional data used to estimate missed cases).

### Data collection

Data collection began on March 6^th^ and ended on April 30^th^ 2014 (total duration 8 weeks). During this 8-week period, all participating practitioners prospectively reported the patients they had seen and who met the following inclusion criteria: 18 years old and over, meeting a diagnosis of psychotic disorder according DSM-IV-TR (i.e. codes 295.xx, 297.x, 298.x) [20] and receiving an antipsychotic treatment prescribed during the consultation. The last criterion (prescription of an antipsychotic during the consultation) was added for two reasons. Firstly, to avoid including subjects currently in remission. Secondly, to avoid counting the same subject more than once, given that subjects were in contact with several physicians during this study period (e.g. a psychiatrist prescribing the psychopharmacological treatment, another psychiatrist supervising psychotherapy at the day-care centre and their GP managing care for medical/somatic problems).

The forms used to report the cases comprised inclusion (see above) and exclusion criteria (e.g. symptoms caused by the effects of a substance, a general medical condition, or a mood disorder), clinical (e.g. positive and negative symptoms, age of onset) and socio-demographical data (e.g. age, sex, month, year and country of birth, an area code for the address). The forms were anonymous, containing no personal details, such as name, address, which could lead to the identification of a subject. The private practice practitioners received the equivalent of a consultation fee for each case reported.

During the survey period, weekly contact was made with the psychiatrists in the public sector and bi-monthly with those in private practice in order to remind them about the study, thereby reducing the risk of errors and missing data.

### Additional data used to estimate missed cases

Not all cases could be identified. Before study onset, the possible reasons for missing cases were analysed. Based on this analysis, we designed the study in order to gather the additional information needed to assess the number of missed cases.

Firstly, for cases seen by their physicians at intervals longer than 8 weeks, the study period was too short for their inclusion. Among these subjects, only a fraction, in inverse proportion with the interval between two appointments, was reported. In order to calculate the number of subjects lost for this reason, information was collected about the interval length between two appointments with their physician. When this interval was larger than the period of the study, we counted each case reported not as one but as the number of weeks between appointments divided by 8 (the duration, in weeks, of the study). For example: of the subjects seen by their prescribing physicians every 12 weeks, it was expected that only 2/3 would be reported, given that the study duration was only 2/3 of the interval between appointments. To account for the 1/3 of subjects not reported in this scenario, the number of subjects seen at 12 week intervals was multiplied by 12/8.

A second cause for missing cases was due to private practice physicians not agreeing to participate in the whole study. To account for this source of lost cases, we calculated several indexes, based on the data from private practice physicians with those that participated in the whole study (GPs and psychiatrists). The first index reflected the proportion of cases actually reported compared with the cases expected before the study (based on the physician’s own estimate); the other two indexes were the mean number of cases reported by a GP or by a psychiatrist. The first index was used for physicians who had given an estimation of the number of cases (before the study) and the two other indexes were for the physicians for whom this prior estimation was not available.

A third possible cause for missing cases is represented by errors in case reporting, i.e. incomplete reporting by the physicians that participated in the study. To assess the number of subjects missed for this reason, we conducted a leakage study. For feasibility reasons, the study was limited to the outpatient clinic - the main provider of cases. Six months after the study took place, each of the psychiatrists working at the outpatient clinic received a list with the names of all the patients they saw in the period of the study. The psychiatrists were instructed to indicate the patients that had a diagnosis of psychotic disorder and, when in doubt (e.g. between a diagnosis of bipolar disorder with psychotic symptoms and a schizo-affective disorder), to consult the case file.

Finally, we estimated that some of the subjects diagnosed with psychosis were treated outside the city of Créteil, thereby escaping reporting procedures. There was no means to exactly assess the number of subjects lost for this reason. However, the figures for the patients that lived outside Créteil and were treated in Créteil were available. Given that Créteil is well equipped for treating psychotic patients, it was thought reasonable to assume that the number of subjects treated outside Créteil will be (as a maximum estimation) equivalent of the number of subjects from outside Créteil treated in Créteil.

### Prevalence calculation

For each of the prevalence estimates reported, the numerator was the number of cases counted and/or estimated and the denominator the total adult population of the catchment area. All prevalence data are reported per 1000 at risk people and with 95 % confidence intervals (CI). Data is also provided for the prevalence in subgroups defined by gender and different age-bands. Finally, the prevalence based on shorter periods of time (1 to 7 weeks) was also calculated, in order to estimate the minimum period needed for a reliable prevalence estimation. To this end, the number of subjects missed because the time period was too short was estimated, using the method described previously (Methods, Additional data used to estimate missed cases, second paragraph), with these then added to the number of subjects actually reported. All denominator data were from the last available census in 2011 [19].

### Ethical approval

The relevant Regional Ethical Committee (Comité de Protection des Personnes – CPP Ile de France VI) examined and approved the study protocol (project number 2011-A01209-32) in accordance with the Helsinki Declaration.

Written consent was not requested because the Ethical Committee agreed that, for ethical reasons, it was important to preserve anonymity of the subjects. Thus, all data sent to the researchers by the treating psychiatrists were anonymous and patients were not in contact with the research team.

## Results

### Participants

**Fig. 1:**
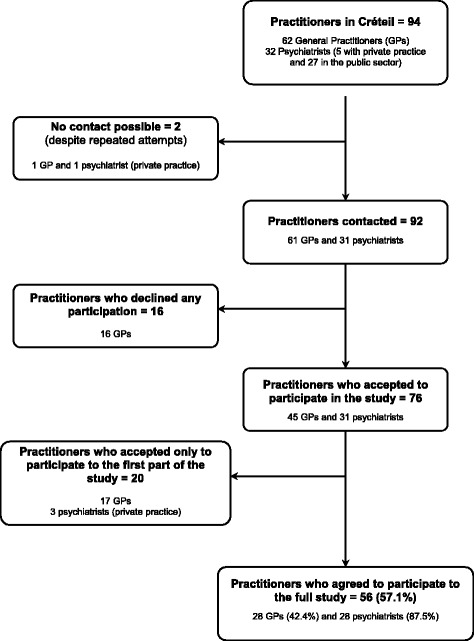
Practitioners present in the catchment area and their participation in the study

All 27 psychiatrists belonging to the public sector participated in the study. Among the private practice psychiatrists, one could not be contacted, 3 only estimated the number of patients treated for psychosis and one participated in the whole study. For GPs, one could not be reached, 16 refused to participate, 17 provided only an estimation of the number of cases and 28 participated in the whole study (for details see Fig. 1).

### First part of the study - estimation of number of cases before the inclusion period

Before the beginning of the inclusion period, forty-four private practice practitioners (among them 3 psychiatrists) provided an estimation of the number of patients that met the inclusion criteria. The total number of expected cases over a two-month period for these practitioners was of 71. The mean number per practitioner was 1.6 (95 % CI 0.81-2.42) and the median number 0.0. Among these practitioners, 28 also participated in the second part of the study. For these practitioners, the mean number of cases anticipated was 2.0 (95 % CI 0.84 - 3.16) and the median 1.0. This was not significantly different from the number of cases expected by the non-participating physicians (mean = 0.94, 95 % CI 0.14-1.74).

### Number of cases reported

**Fig. 2:**
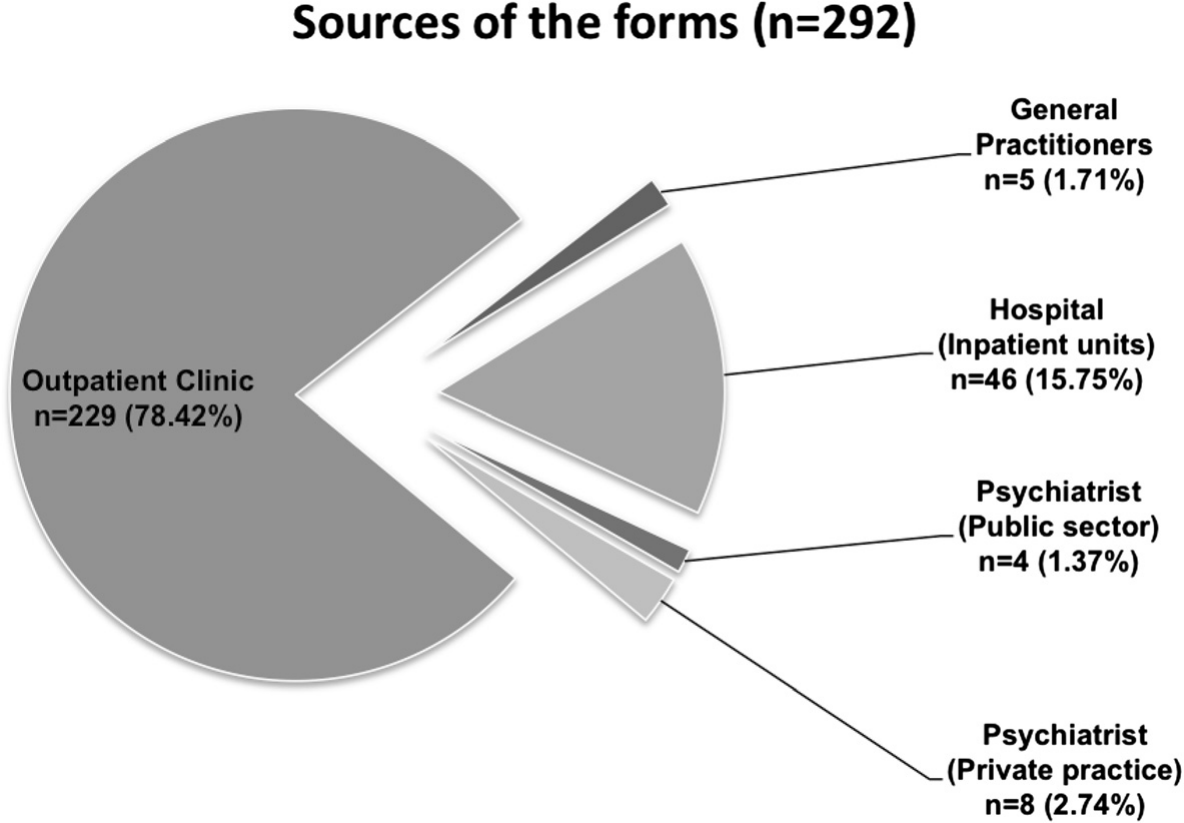
Source of the reported cases

We received a total of 292 forms. Six of them were duplicates (same age, gender, area code, etc.); and 35 were from subjects outside Créteil. Most of them (229; 78.4 %) came from the outpatient clinic (for details see Fig. 2). The 28 practitioners who estimated the number of subjects seen in their practice sent back 5 forms. The leakage study identified 8 more subjects having the inclusion criteria and living in Créteil (i.e. 3.5 % of the cases initially reported) and 5 subjects living outside Créteil.

### Prevalence calculations

#### Prevalence based on cases reported

**Fig. 3:**
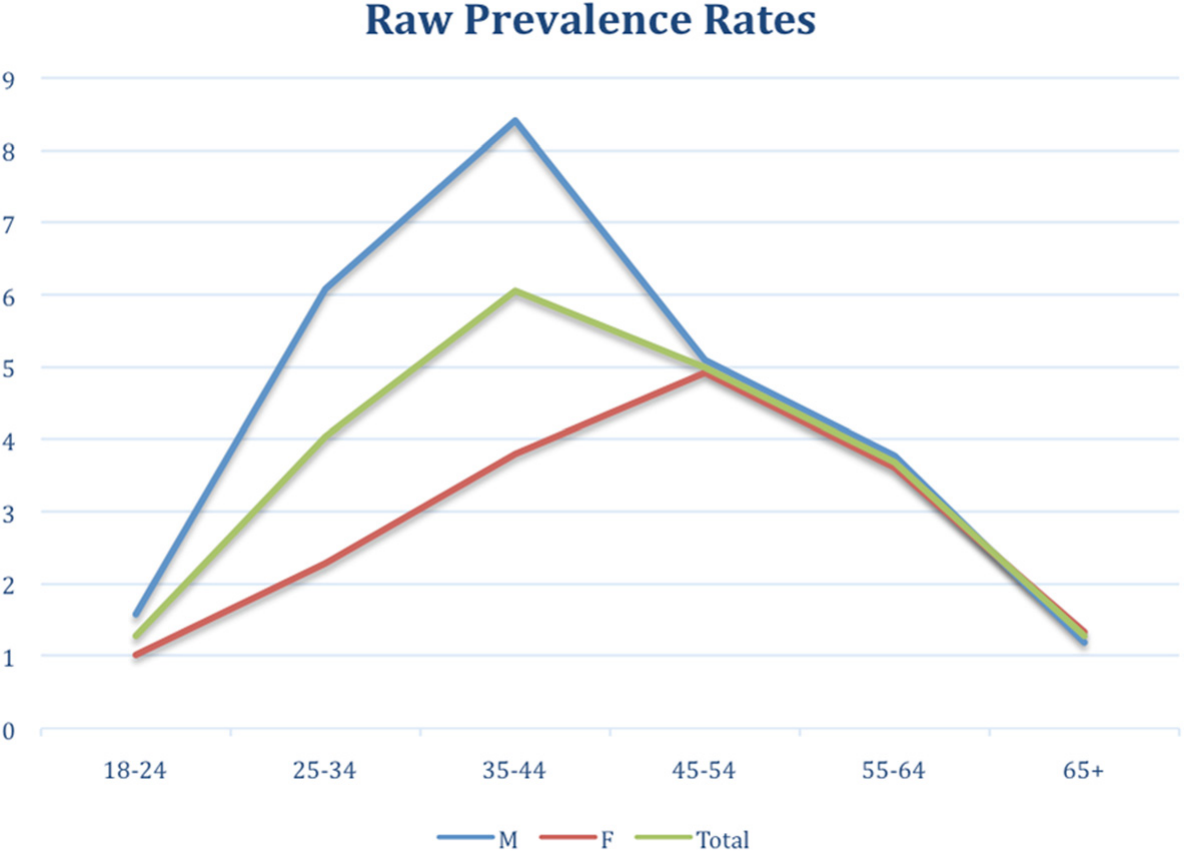
Raw prevalence (per 1000) by sex and age-bands

Based on the reported cases and after eliminating the duplicates, the global prevalence was 3.72 per 1000. The prevalence was higher in men (relative risk (RR) = 1.68) and in the 35–44 years age-band (RR compared with all other age-bands = 1.93) and lower in the extreme age bands, i.e. 18–24 (RR = 0.31) and 65 and older (RR = 0.31) (for details, see Table 1 and Fig. 3).

**Table 1:** Raw prevalence rates by sex and age-bands

Age Bands	F (95 % CI)	M (95 % CI)	Total (95 % CI)
18-24	1.01 (0.13-1.90)	1.57 (0.41-2.73)	1.28 (0.55-2.00)
25-34	2.27 (1.19-3.35)	6.08 (4.18-7.98)	4.03 (2.98-5.08)
35-44	3.80 (2.28-5.31)	8.41 (6.11-10.71)	6.05 (4.69-7.42)
45-54	4.92 (3.22-6.62)	5.08 (3.20-6.95)	4.99 (3.73-6.25)
55-64	3.60 (1.94-5.26)	3.77 (1.98-5.55)	3.68 (2.46-4.89)
65+	1.34 (0.41-2.27)	1.18 (0.15-2.22)	1.27 (0.58-1.97)
Total	2.87 (2.35-3.39)	4.71 (3.95-5.47)	3.72 (3.26-4.18)

#### Estimate of the prevalence of cases treated in Creteil

Only 10 subjects were seen at intervals longer than 8 weeks (mean interval for these subjects = 10.65 weeks). This gives an estimate of 3.31 for subjects lost for this reason.

For private practice practitioners who participated in the whole study, the number of subjects actually reported (*N* = 5) divided by the number expected (*N* = 56) was of 0.09. The practitioners who participated only in the first step of the study estimated the number of subjects that they treated for psychosis to be 15. As such, the number of subjects lost due to these practitioners not participating in the whole study was estimated at 1.35. The mean number of subjects reported by the GPs participating in the whole study was 0.18. As such, it was estimated that the number of subjects for the 17 GPs not participating in the study to be 3.06. For the psychiatrist that did not participate in the study, we estimated the number of patients based on the number of patients (without duplicates) reported by the private psychiatrists that did participate in the whole study, i.e. 7.

Adding all these numbers, the number of forms that we expected to receive if the study continued for a longer period of time and if all practitioners had agreed to participate, was 265.69.

Finally, the leakage study revealed that the proportion of cases not reported was 3.49 %. When the same proportion was added to the previous estimate, we obtained a total number of cases of 274.96 and a prevalence of 4.08 per 1000 (95 % CI 2.30-5.85).

#### Estimate of the prevalence of treated psychosis among the population of Creteil (including cases treated outside Créteil)

**Fig. 4:**
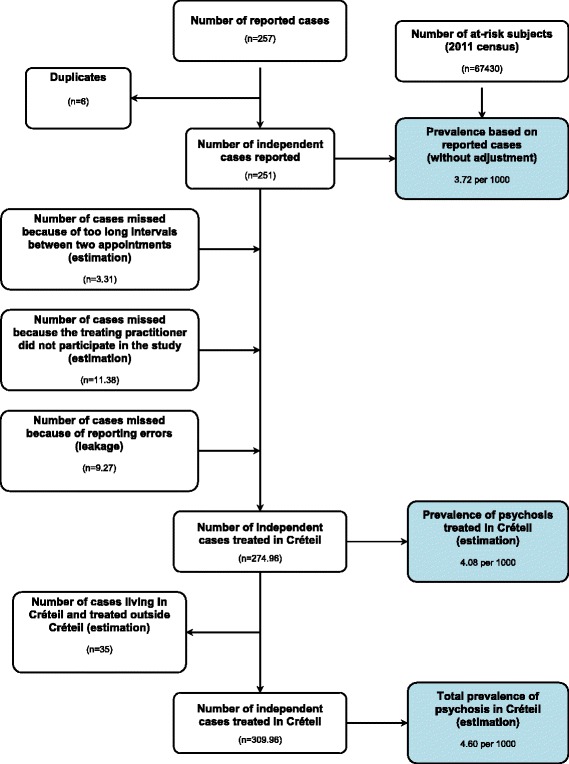
Estimation of the number of cases and prevalence of treated psychosis in Créteil

This estimate is based on the number of subjects with psychosis from Créteil and treated in or outside Créteil. The later number was estimated to be equivalent to the number of patients living outside Créteil, but treated in Creteil, i.e. 35. (30 cases living outside Créteil identified during the data collection plus 5 cases identified during the leakage study). As such, the estimate of the total prevalence of treated psychosis in Creteil is 4.60 per 1000 (95 % CI 2.71-6.48) (see Fig. 4 for more details).

### Estimation of the raw prevalence at shorter intervals

**Fig. 5:**
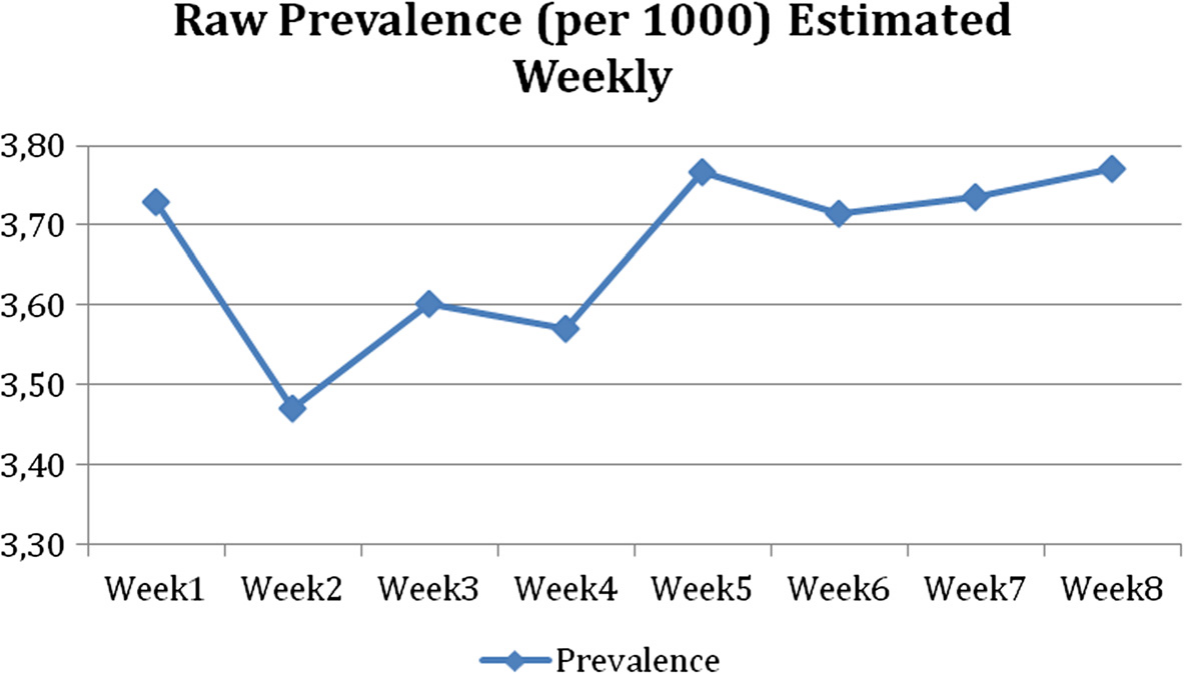
Raw prevalence estimated weekly

Figure 5 shows the curve of the prevalence estimated weekly, based on reported cases. For the general estimate of prevalence, a 5 week period seems appropriate. A 6 week period seems more appropriate, if more details are required, such as prevalence broken down by gender and age bands (further details available from the authors on request).

## Discussion

In this study, we report data on period prevalence (8 weeks) of treated psychotic disorders in an urban area in France. After adjustment for potential sources of lost cases, the prevalence of treated psychosis was estimated to be 4.60 per 1000. Most of the cases were outpatients (79.79 %) that were treated by psychiatrists (98.29 %) in the public sector (95.55 %).

Taking into account the limitations of the study in regard to duration (i.e. adjusting for the 8 weeks of the study period), location (adjusting for patients receiving their treatment outside Créteil), number of participating practitioners or the inherent imperfect reporting of cases (leakage study), the estimated prevalence (3.72 per 1000) increased by more than 20 %. In a systematic review, Goldner and colleagues found a period prevalence for schizophrenia and related disorders of 6.0 per 1000 (95 % CI 3.8-9.1). Our figures are in the lower half of this confidence intervals, which may be due to the shorter study period (8 weeks vs. 1 year) and/or to the inclusion of only treated cases in the study reported here [2].

An interesting finding is indicated by the shape of the graph of prevalence by age-bands. Based on the assumption of lifetime disorders, it was expected that the prevalence would be larger in later age bands, with the possible exception of the last age band when, due to an excess mortality in patients with psychosis and small incidence numbers, a slower decline in prevalence would have been expected. Instead, we observed the highest prevalence of psychosis in the 35–45 year age band. The lower prevalence in subsequent age bands could be due to excess mortality in psychotic patients, or to secular trends for prevalence (increases in incidence/prevalence in more recent years). However, given the amplitude of this decline (almost 40 % between the 35–45 and the 55–65 age bands), it seems unlikely that such explanations could completely account for the observed trend, instead suggesting that psychosis, once diagnosed, is not a life-time treated disorder.

Incidence rates from the same area are of 0.22 per 1000 subjects*year [21], therefore suggesting a mean time of 15 to 20 years of treated psychosis. This seems to be consistent with epidemiological data on schizophrenia summarized by Saha and colleagues for the prevalence [7] and McGrath and colleagues for incidence (0.15 per 1000 population*year median incidence for schizophrenia), as well as being consistent with studies [22] that reported both incidence and prevalence data (summarized by Saha et al. [8]), suggesting the same (mean) interval of follow-up.

Various reasons for this could be suggested: a mixture of short lived disorders and chronic, life-long disorders, chronic patients moving preferentially out of the area (for example to specialized care facilities), milder clinical forms that are no longer treated, or complete recovery.

Based on longitudinal and long-term follow-up studies, the annual rate of recovery was estimated by Saha and colleagues to be 1.37 % [8] suggesting that, although recovery could explain part of the observed data, it is probable that it is not the sole explanation. Data from a more recent meta-analysis of studies on recovery from schizophrenia and related disorders [23], found a similar annual recovery rates (1.4 %). However, this study also suggested that this rate diminishes with the duration of the disorder [23]. At a constant 1.4 % annual recovery rate (and without any new cases), the number of subjects not recovered after 20 years will be around 75 % of the initial number. We found a significantly larger difference (40 %) between the third (35–44) and fifth (55–65) age-bands suggesting that, although recovery could explain a significant part of the observed data, it is unlikely that it is the sole explanation. Finding the explanation for this observation deserves further studies, especially, longitudinally derived data.

As mentioned in the introduction, there are very few studies on the prevalence of psychotic disorders in France. The most recent data are from the French Mental Health in General Population (MHGP). The figures for psychotic disorders in this study were 2.7 % comprised of 0.7 % single psychotic episodes and 2 % recurrent psychotic disorders [15]. Our figures are smaller. Part of the difference could be due to the MHGP study reporting lifetime prevalence, whereas we reported period prevalence. Another important difference is that we reported treated psychosis and they reported all cases, treated or not. Finally, the difference may be due to some of the subjects in the MHGP study not having been diagnosed with a psychotic disorder according to DSM criteria, given the lack of duration and/or impairment criteria in that study. Although the MINI showed excellent psychometric qualities in a validation study conducted in a sample comprised of mainly psychiatric patients [17], its diagnostic accuracy, especially specificity, in the general population has, to our knowledge, not been assessed.

There are several limitations in our study that have to be acknowledged. Some of the physicians did not participate in the study. Although we tried to estimate the number of cases missed for this reason, there are uncertainties about the exact number of cases. We were surprised by the difference between the number of cases anticipated by the practitioners and the actual number of cases reported. In retrospect, one possible explanation is that they included in their estimation, those patients suffering from psychosis but for whom antipsychotic treatment was being prescribed by another physician. It is of note that the two methods of estimating the number of cases lost due to the GP not participating in the whole study (i.e. based on the mean number of subjects reported by participating GP or based on the number of subjects anticipated) lead to similar results. However, not taking into account the number of cases anticipated by the psychiatrists that participated only in the first step (and consider that they had the same number of patients as the only private practice psychiatrist that did participate) would have resulted, in our view, in an overestimation. Using this method, the number estimated would have been of 21 in considerable contrast to their estimation of no subject for which they prescribed antipsychotic treatment (in their practice they mostly provided psychotherapy).

Another limitation of the study was that there was no information from the non-participating physicians and therefore, we cannot be sure that data from the participating practitioners accurately represent all cases. However, more than 80 % of the physicians from the area participated in at least to the first part of the study. The physicians that did not participate were, for the most part, GPs, which was the category of physicians that reported the smallest number of cases. As such, the number of cases lost for this reason is probably small.

In order to estimate the number of cases lost because errors in reporting, a leakage study was carried out. However, this study was limited to psychiatrists from the outpatient clinic and it is uncertain that extending the findings from the leakage study to all cases would have been appropriate. Nevertheless, the leakage study covered a large majority of reported cases (almost 80 %), indicating that even if the leakage study results for the remaining physicians had been different, it is probable that the impact on the overall estimation of cases would have been limited.

We had no means to exactly assess the number of subjects living in the area but treated outside Créteil. Créteil is in close proximity to other urban areas (including Paris), but is also well equipped for treating patients with psychotic disorders. As such, although it is probable that there is a significant number of subjects that are treated outside their home town, it is also probable that for Créteil this number does not exceed the number of patients treated here but living outside Créteil.

Another limitation of our study is that we did not record specific diagnoses. This limits our capacity to compare our results with those in the literature, as most previous studies reported data only for schizophrenia. Our method of identifying cases relied on the presence of characteristic symptoms, which we thought would be more reliable and comparable across physicians than specific diagnoses, especially for cases identified by GPs.

One important point to note is the reporting here of the prevalence of treated psychosis which is, by definition, smaller that the prevalence of psychosis as a whole. The number of untreated cases cannot be estimated from this study. We also made the choice to limit our study to psychotic disorders as defined by DSM-IV-TR, also called non-affective psychoses. As a consequence, our findings do not apply to all disorders with psychotic features, such as mood disorders with psychotic features (sometimes referred as "affective psychoses").

Finally, our study took place in a highly urbanized site. It is not clear if prevalence of psychotic disorders is influenced by urbanization. Although the review by Saha and colleagues did not find significant differences in prevalence according to the degree of urbanisation, this contrasts with consistent data showing significantly higher incidence of psychotic disorders in urban areas [7, 24, 25]. Therefore, at this point, our results cannot be generalized to the whole French population, especially to more rural sites.

There are several potential implications of our results. Firstly, they underscore the need to reinforce mental health services that aim to provide adequate and specific care to young patients. Secondly, they suggest that more research is needed to understand the sizable difference in prevalence between subjects in their 40s and subjects in the 55–64 age-band. It is essential that future investigations should explore the outcome, and the need for psychiatric care, of patients that are no longer in contact with mental health services. This may require a raising of the awareness of this phenomenon and is likely to necessitate a better collaboration between psychiatrists and GPs.

## Conclusions

The estimates in our study are in line with expected numbers that are based on studies conducted in other countries. In our study, careful consideration of causes of missed cases and gathering of complementary data (including a leakage study) aimed at estimating the number of lost cases, resulted in a significant increase in the estimated prevalence (more than 20 %). This indicates that careful consideration of methodological limitations, prior to study commencement, is essential in order to obtain valid prevalence estimates.

An interesting finding, deserving further investigations, is the marked differences between age specific prevalence rates, with surprisingly relatively low figures in the 55–64 age-band, a finding that is more pronounced in men. Understanding the origin of the difference between prevalence in the 35–44 and 55–64 age-bands may contribute to the provision of new public health measures that aim to reduce mortality in this population, as well as improving the identification and treatment of patients that are still symptomatic but no longer in treatment.
